# Dose Escalation of Biologics in Biologic-Naïve Patients With Ulcerative Colitis: Outcomes From the ODESSA-UC Study

**DOI:** 10.1093/crocol/otad061

**Published:** 2023-11-16

**Authors:** Sabyasachi Ghosh, Niranjan Kathe, Kandavadivu Umashankar, Kirti Mirchandani, Arunima Hait, Riyanka Paul, Ninfa Candela, Tao Fan

**Affiliations:** Takeda Pharmaceuticals U.S.A., Inc., Lexington, MA, USA; Complete HEOR Solutions, Chalfont, PA, USA; Takeda Pharmaceuticals U.S.A., Inc., Lexington, MA, USA; Complete HEOR Solutions, Chalfont, PA, USA; Complete HEOR Solutions, Chalfont, PA, USA; Complete HEOR Solutions, Chalfont, PA, USA; Takeda Pharmaceuticals U.S.A., Inc., Lexington, MA, USA; Takeda Pharmaceuticals U.S.A., Inc., Lexington, MA, USA

**Keywords:** ulcerative colitis, biologics, dose escalation, vedolizumab, biologic-naïve

## Abstract

**Background:**

Dose escalation of biologics may regain treatment response in patients with ulcerative colitis (UC). However, dose escalation rates and associated outcomes and costs are not well characterized in biologic-naïve patients receiving antitumor necrosis factor-alpha (anti–TNF-α) treatments, such as infliximab or adalimumab or vedolizumab.

**Methods:**

ODESSA-UC, a retrospective cohort study investigating dose escalation in patients with UC who had received first-line biologics, used data from IBM MarketScan databases. Adults with UC and ≥1 claim for an index drug (adalimumab, infliximab, or vedolizumab) were eligible. A Cox proportional hazards model was used to evaluate the adjusted rate of dose escalation. Logistic regression was used to evaluate the odds of experiencing adverse outcomes (corticosteroid use, infection, sepsis, or inflammatory bowel disease–related hospitalization) and incurring index drug costs.

**Results:**

A year after the start of maintenance, a lower proportion of patients experienced dose escalation with vedolizumab (22.3%) than adalimumab (43.0%). The dose escalation risk was significantly higher for infliximab (hazard ratio [HR], 1.894; 95% confidence interval [CI], 1.486–2.413) and adalimumab (HR, 2.120; 95% CI, 1.680–2.675) than for vedolizumab. The odds of experiencing an adverse outcome after dose escalation were higher for anti–TNF-α treatments than for vedolizumab (odds ratio, 2.052; 95% CI, 1.200–3.507). Index drug costs after dose escalation were lowest for vedolizumab.

**Conclusions:**

Patients with UC receiving vedolizumab had a lower risk of dose escalation and lower subsequent costs than patients receiving anti–TNF-α treatments. Our study demonstrates the possible clinical and economic implications of dose escalation.

## Graphical Abstract

**Graphical Abstract:**
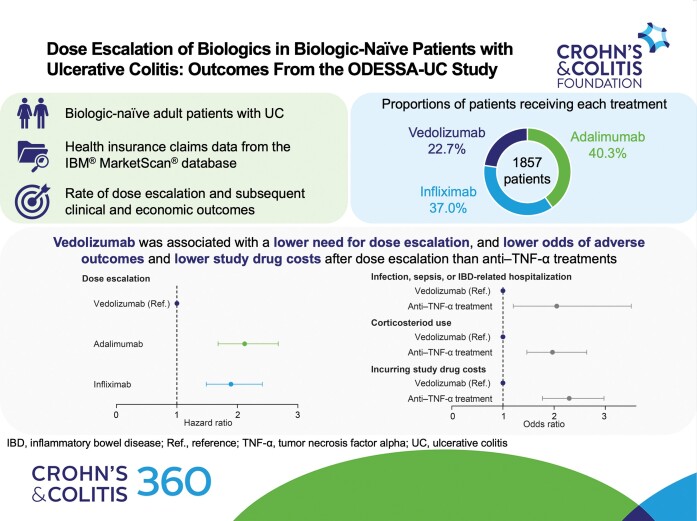

**Key Messages**

1. What is already known?

Most data on rates and outcomes of dose escalation are limited to antitumor necrosis factor-alpha treatments, with reported rates of dose escalation varying widely.

2. What is new here?

Adjusted rates of dose escalation, adverse outcomes, and costs after dose escalation are lower for vedolizumab than for antitumor necrosis factor-alpha treatments.

3. How can this study help patient care?

This study may help clinicians and payers to understand the clinical implications of dose escalation and its impact on healthcare resource use.

## Introduction

Ulcerative colitis (UC) is a chronic inflammatory disease characterized by inflammation of the rectum and colon.^1^ The disease often follows a relapsing and remitting course, with periods of active disease and symptom flares alternating with periods of disease remission.^1–3^

Corticosteroids may be used for the acute management of UC; however, they are not recommended for use as a maintenance treatment owing to the various side effects associated with their long-term use.^2,4^ The aim of treatment for UC is to therefore induce and sustain corticosteroid-free remission, alongside the avoidance of hospitalization and surgery as well as other disease-related adverse outcomes.^2^

Biologics, such as the antitumor necrosis factor-alpha (anti–TNF-α) treatments adalimumab and infliximab, or the α4β7 integrin inhibitor vedolizumab, are recommended for treating patients with moderate-to-severe UC.^2^ Despite these options demonstrating efficacy, patients may experience primary nonresponse, whereby they do not respond to their initial treatment, or secondary loss of response, in which they lose response to their prescribed biologic treatment during maintenance.^5,6^ Patients who experience secondary loss of response may require treatment plan modifications, such as dose escalation, to recapture response.^5^ Previous research has demonstrated that, for anti–TNF-α treatments, secondary loss of response may result from inadequate dosage either with or without the presence of antidrug antibodies.^7,8^

Dose escalation of biologics has been shown to increase healthcare costs in patients with UC.^9^ Reported rates of dose escalation for patients receiving anti–TNF-α treatments vary widely, while data on the rates of dose escalation in patients receiving vedolizumab are more limited.^6^ Real-world studies that compare the frequency of dose escalation in biologic-naïve patients with UC receiving anti–TNF-α treatments or vedolizumab are lacking.^6^ In addition, there are limited data on adverse events and economic outcomes after dose escalation, as well as the time to dose escalation.^6,9,10^ Such data may enhance stakeholders’ understanding of the clinical implications of dose escalation and associated healthcare resource use.

Here, we report the real-world rates of dose escalation, as well as index drug costs, and the proportion of patients experiencing adverse outcomes following dose escalation in adult patients with UC who received adalimumab, infliximab, or vedolizumab as first-line biologics.

## Materials and Methods

### Objectives

The ODESSA-UC (real wOrld Dose EScalation and outcomeS with biologics in IBD pAtients with UC) study aimed to evaluate dose escalation in biologic-naïve patients with UC. The primary objective was to compare the proportions of patients whose dose was escalated across biologic treatment groups in the first 6 and 12 months after the start of maintenance treatment, and over the entire maintenance period. The exploratory objectives were to compare the hazard rate of dose escalation for vedolizumab with anti–TNF-α treatments in the 12 months after the initiation of maintenance dosing, and to compare adverse outcomes and costs after dose escalation across treatment groups.

### Study Design

**Figure 1.**
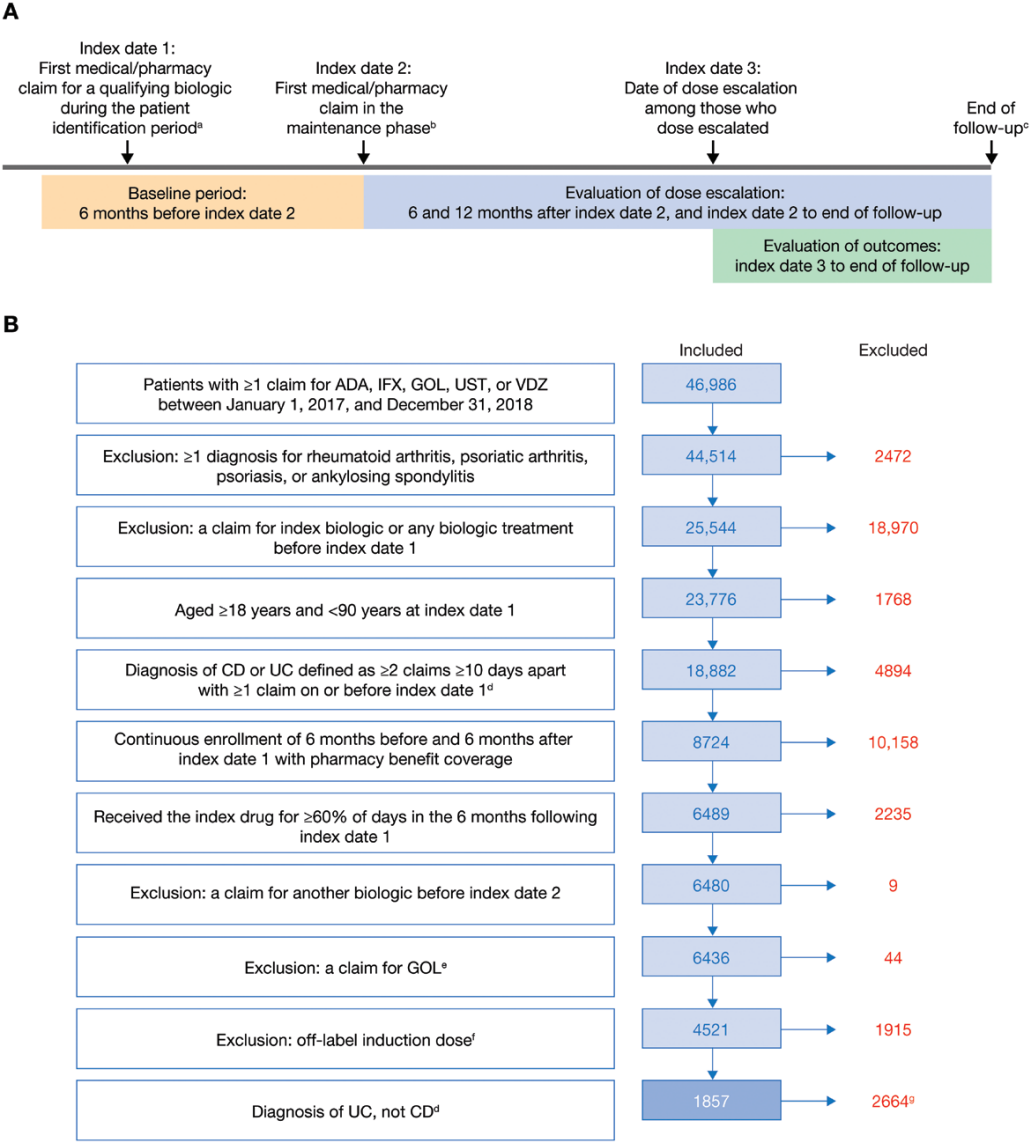
(A) Study design and (B) cohort size, inclusion and exclusion criteria, and patient attrition. Study design (A) consisted of a baseline period, evaluation of dose escalation, and evaluation of outcomes. Exclusion and inclusion criteria determined the final cohort size (B). ^a^Patient identification period: January 1, 2017, to December 31, 2018. ^b^The maintenance phase began on the date of the third (adalimumab) or fourth (infliximab or vedolizumab) pharmacy claim after index date 1. ^c^Whichever came first of end of enrollment, switching date, discontinuation date, or June 30, 2020. ^d^For patients with diagnoses of both CD and UC, the number of consecutive diagnoses during the baseline period was used to classify patients. ^e^As a consequence of low patient count, patients treated with GOL were excluded although they were initially intended to be included in the analyses. ^f^Defined as a higher dose or fewer days than the recommended prescribing regimen for induction. ^g^The results of the CD cohort are reported separately. Although included in the Crohn’s disease cohort, patients who received ustekinumab were excluded from the ulcerative colitis cohort since approval of ustekinumab for ulcerative colitis was only obtained in October 2019; thus, not enough data would be available for analysis. ADA, adalimumab; CD, Crohn’s disease; GOL, golimumab; IFX, infliximab; UC, ulcerative colitis; UST, ustekinumab; VDZ, vedolizumab.

ODESSA-UC was a retrospective observational study conducted using administrative claims data from IBM MarketScan databases. The study design is shown in Figure 1A. The patient identification period was January 1, 2017, to December 31, 2018. The date of the first medical/pharmacy claim for a qualifying biologic (termed “index drug”) during the patient identification period was used to define index date 1, while index date 2 was defined as the date of the first medical/pharmacy claim in the maintenance phase. The first medical/pharmacy claim in the maintenance phase was the date of the third claim for adalimumab from index date 1 or the date of the fourth claim for infliximab or vedolizumab from index date 1. The baseline period was the 6-month period before index date 2. Index date 3 was defined as the date of dose escalation among patients whose dose was escalated. The follow-up period was defined as the time from index date 3 to whichever came first: end of enrollment, switching date, discontinuation date, or June 30, 2020. End of enrollment was defined as the first occurrence of a gap of more than 45 days in continuous enrollment. Switching was defined as the first instance of a claim for a qualifying biologic other than the index drug. Discontinuation was defined as the first instance of a period of 60 days between the last day of supply on a claim and start of the subsequent claim.

### Data Source

This study used data from 2 IBM MarketScan databases (IBM Watson Health, Hershey, PA). The IBM MarketScan Commercial Claims and Encounters Database consists of adjudicated inpatient, outpatient, and pharmaceutical claims from over 50 million employees and their dependents, annually, who are covered by employer-sponsored private health insurance in the United States. The IBM MarketScan Medicare Supplemental and Coordination of Benefits Database includes inpatient, outpatient, and pharmaceutical claims for Medicare-covered retirees and their dependents, with employer-sponsored supplemental insurance.

### Study Population

Patients were eligible for inclusion if they met the following criteria: at least 1 claim for adalimumab, infliximab, golimumab, ustekinumab, or vedolizumab between January 1, 2017, and December 31, 2018; at least 18 but less than 90 years of age at index date 1; and at least 2 claims at least 10 days apart for UC or Crohn’s disease, with at least 1 claim on or before index date 1 (Figure 1); continuous enrollment of 6 months before and 6 months after index date 1 with pharmacy benefit coverage; and at least 60% of days covered during the first 6 months after index date 1. The proportion of days covered was calculated using the following formula:


$$\frac{\text{Days in follow-up period covered by claims for a qualifying treatment}}{\text{Number of days in follow-up period }}$$

For intravenous medications, the recommended dosing schedule was used to inform the number of days with claims.

Patients were excluded from the analyses if they met any of the following criteria: Claims for any biologic in the 6 months before index date 1; diagnosis of ankylosing spondylitis, psoriasis, psoriatic arthritis, or rheumatoid arthritis in the baseline period (Table S1); an equal number of claims for Crohn’s disease and UC; or a combination or overlap of two or more biologics after index date 1. Although included in the Crohn’s disease cohort, patients who received ustekinumab were excluded from the UC cohort because approval of ustekinumab for UC was only obtained in October 2019^11^; thus, not enough data would be available for analysis. Additionally, patients with a claim for golimumab and patients with claims for an off-label induction dose, defined as a higher dose or fewer days than the recommended induction prescribing regimen for their biologic, were excluded.

Eligible patients were assigned to a mutually exclusive cohort of Crohn’s disease or UC based on the number of consecutive diagnoses during the baseline period. To minimize bias due to misclassification, patients with at least 3 consecutive Crohn’s disease or UC claims were assigned to Crohn’s disease or UC cohorts, respectively. The inclusion and exclusion criteria were used to determine the final cohort size. Here, we present the results for the UC cohort.

### Variables

Baseline variables were captured using data recorded 6 months before index date 2. Demographics included age, sex, and US region. Baseline clinical characteristics included age-adjusted Charlson Comorbidity Index (ACCI) score, comorbid conditions, infections, disease extent, all-cause hospitalization, inflammatory bowel disease (IBD)-related hospitalization, UC-related surgery, and concomitant use of immunomodulators, immunosuppressive agents, or anti-inflammatory agents (6-mercaptopurine, azathioprine, balsalazide, cyclosporine, leflunomide, lenalidomide, mesalamine, methotrexate, mycophenolate mofetil, olsalazine, sirolimus, sulfasalazine, tacrolimus, thalidomide, and thioguanine).

### Outcomes

Dose escalation was defined as an increase of at least 20% in the average daily dose relative to the expected daily dose based on the approved prescribing information for UC (Table S2).^12–14^ Given that infliximab uses weight-based dosing, the expected daily dose was defined based on the initial strength dispensed.^12^ Average daily dose was calculated per the following formula as defined in a recent database study of patients with IBD^15^:


$$\frac{\text{Actual quantity dispensed }\left(mg\right)}{\text{The number of days between the fill dates of the two adjoining claims}}$$

Exploratory outcomes were evaluated from index date 3 to the end of follow-up for each treatment cohort. Exploratory outcomes included: the proportion of patients with infection or sepsis; the proportion of patients with IBD-related hospitalizations; the proportion of patients with corticosteroid use; and index drug costs. The presence of infection or sepsis was identified using International Classification of Diseases, 10th Edition (ICD-10) codes (Table S3). IBD-related hospitalizations were identified using inpatient admissions and an ICD-10 diagnosis code for IBD (Table S1) in any position (diagnosis principal [PDX], diagnosis 1 [DX1], diagnosis 2 [DX2], diagnosis 3 [DX3], diagnosis 4 [DX4], and diagnosis 5 [DX5]). Corticosteroid use was identified using Therapeutic Detail Codes and Healthcare Common Procedure Coding System codes for betamethasone, budesonide, cortisone, dexamethasone, hydrocortisone, methylprednisolone, prednisone, prednisolone, and triamcinolone. Index drug costs were defined as the medical and pharmacy costs associated with the index drug. Costs were identified from claims identified using National Drug Codes and Healthcare Common Procedure Coding System codes.

### Statistical Analysis

Biologics included in this analysis were adalimumab, infliximab, and vedolizumab. Baseline demographics and clinical characteristics were analyzed using descriptive statistics, with analysis of variance and Chi-square tests performed to compare differences in baseline characteristics between treatment groups for continuous variables and categorical variables, respectively. Fisher’s exact test was used for groups with fewer than 5 patients.

Covariates for adjusted models included age, sex, Charlson Comorbidity Index, anxiety or depression, all-cause hospitalization, IBD-related surgery, IBD-related concomitant medication, infection, disease location, and complications such as fistula or abscess.

The proportions of patients whose dose was escalated were analyzed using descriptive statistics. A Cox proportional hazards regression model, adjusted for differences in covariates between the groups, was used to compare the rates of dose escalation between vedolizumab and the anti–TNF-α treatments in the 12 months after initiation of maintenance, with time defined as: index date 3−index date 2  for patients whose dose was escalated, and: minimum(end of follow-up,  (index date 2+365))−index date 2 for patients whose dose was not escalated.

Kaplan–Meier analysis was used to evaluate time to dose escalation in the 12 months after initiation of maintenance. Logistic regression, adjusted for covariates, was used to evaluate the likelihood of a composite outcome of infection, sepsis, or IBD-related hospitalization, and the likelihood of corticosteroid use, from index date 3 to the end of follow-up. Infection, sepsis, and IBD-related hospitalization were originally planned as separate analyses but were combined owing to low event rates.

Adjusted generalized linear models were used to conduct multivariable analysis on index drug costs from index date 3 to the end of follow-up. The 2-part regression generalized linear model consisted of a binary logit model, used to estimate the likelihood of incurring any index drug cost after dose escalation, and a gamma distribution and log link-based model, used to estimate the ratio of expected index drug costs conditional on patients having a positive cost (ie, patients whose dose was escalated). Index drug costs were calculated only for patients whose dose was escalated; the costs for patients whose dose was not escalated were imputed as zero. Costs were measured from a commercial payer perspective. Gross payments were not used to capture costs owing to missing data between 2019 and 2020. Statistical analyses were carried out using SAS v9.4 or later (SAS Institute, Inc., Cary, NC; 2011).

## Results

### Study Cohort

After applying the patient selection criteria, 1857 eligible patients with UC were identified and included in the analyses (Figure 1B). Of these 1857 patients, 421 (22.7%), 688 (37.0%), and 748 (40.3%) patients received vedolizumab, infliximab, and adalimumab, respectively.

### Baseline Demographics and Clinical Characteristics

Baseline demographics and clinical characteristics stratified by index drug are shown in Table 1. The treatment groups had similar demographics, including age, sex, and US region. Mean age was in the fourth decade for all 3 groups, and each group had a slightly greater proportion of men than women. The US South was the most common region for all groups. There were some differences between the treatment groups with regard to clinical characteristics. At baseline, the number of all-cause hospitalizations and IBD-related hospitalizations varied between the groups (*P* < .0001 for both). A greater proportion of patients who received infliximab were hospitalized than those who received adalimumab or vedolizumab. A greater proportion of patients in the vedolizumab group (4.0%) had cancer than in the adalimumab (1.5%) or infliximab (1.5%) groups (*P* = .0046), while a greater proportion of patients had anemia in the infliximab (19.5%) group than in the adalimumab (11.4%) or vedolizumab (12.6%) groups (*P* < .0001). Claims for concomitant medications (immunomodulators, immunosuppressive agents, or anti-inflammatory agents) also significantly differed between the groups (*P* < .0001), with a greater proportion of patients receiving adalimumab requiring claims than those receiving vedolizumab or infliximab. The proportions of patients receiving each immunomodulator, immunosuppressive agent, or anti-inflammatory agent are presented in Table S4. Regarding disease extent, a lower proportion of patients in the adalimumab (28.6%) group had pancolitis than in the infliximab (35.8%) and vedolizumab (34.7%) groups (*P = *.0093).

**Table 1.** Baseline demographics and clinical characteristics of patients with ulcerative colitis.

Demographic or characteristic	Adalimumab (*n *= 748)	Infliximab (*n *= 688)	Vedolizumab (*n *= 421)	*P* value
Age, years, mean (SD)	41.5 (13.40)	40.9 (14.06)	42.8 (13.33)	.0709
Age range, years, *n* (%)				
18–34	260 (34.8)	252 (36.6)	132 (31.4)	.2961
35–49	246 (32.9)	210 (30.5)	140 (33.3)	
50–64	233 (31.1)	214 (31.1)	137 (32.5)	
≥65	9 (1.2)	12 (1.7)	12 (2.9)	
Sex, *n* (%)				
Male	408 (54.5)	370 (53.8)	220 (52.3)	.7527
Female	340 (45.5)	318 (46.2)	201 (47.7)	
US region, *n* (%)				
Northeast	147 (19.7)	132 (19.2)	66 (15.7)	.0308
North Central	150 (20.1)	186 (27.0)	97 (23.0)	
South	357 (47.7)	274 (39.8)	195 (46.3)	
West	92 (12.3)	94 (13.7)	61 (14.5)	
Other or unknown	2 (0.3)	2 (0.3)	2 (0.5)	
All-cause hospitalizations, *n* (%)				
0	620 (82.9)	483 (70.2)	372 (88.4)	<.0001
1	102 (13.6)	149 (21.7)	41 (9.7)	
>1	26 (3.5)	56 (8.1)	8 (1.9)	
IBD-related hospitalizations, *n* (%)				
0	632 (84.5)	491 (71.4)	377 (89.5)	<.0001
1	96 (12.8)	146 (21.2)	37 (8.8)	
>1	20 (2.7)	51 (7.4)	7 (1.7)	
Comorbidities				
Cardiovascular disease	8 (1.1)	9 (1.3)	1 (0.2)	.1969
Chronic pulmonary disease	61 (8.2)	49 (7.1)	29 (6.9)	.6596
Renal disease	10 (1.3)	8 (1.2)	6 (1.4)	.9229
Liver disease	34 (4.5)	36 (5.2)	23 (5.5)	.7435
Cancer	11 (1.5)	10 (1.5)	17 (4.0)	.0046
Diabetes mellitus	45 (6.0)	44 (6.4)	23 (5.5)	.8183
Rheumatic disease^a^	5 (0.7)	7 (1.0)	3 (0.7)	.7385
Anemia	85 (11.4)	134 (19.5)	53 (12.6)	< .0001
Mental disorders^b^	2 (0.3)	4 (0.6)	3 (0.7)	.4727
ACCI score, mean (SD)	1.3 (1.50)	1.3 (1.61)	1.5 (1.67)	.1078
Immunomodulators, immunosuppressive agents, or anti-inflammatory agents, *n* (%)				
1–60 days covered	63 (8.4)	91 (13.2)	41 (9.7)	<.0001
>60 days covered	685 (91.6)	548 (79.7)	345 (81.9)	
No claims	0 (0.0)	49 (7.1)	35 (8.3)	
Infections, *n* (%)	66 (8.8)	59 (8.6)	33 (7.8)	.8428
Surgery (UC related),^c^*n* (%)	0 (0.0)	2 (0.3)	2 (0.5)	.1258
Disease extent,^d^*n* (%)				
Pancolitis	214 (28.6)	246 (35.8)	146 (34.7)	.0093
Left-sided	56 (7.5)	42 (6.1)	45 (10.7)	.0202
Proctosigmoiditis	42 (5.6)	36 (5.2)	24 (5.7)	.9294
Proctitis	34 (4.5)	28 (4.1)	12 (2.9)	.3599
Other or unspecified	402 (53.7)	336 (48.8)	194 (46.1)	.0284

Abbreviations: ACCI, age-adjusted Charlson Comorbidity Index; IBD, inflammatory bowel disease; SD, standard deviation; UC, ulcerative colitis.

^a^Patients with diagnosis codes for rheumatic disease who were not included in rheumatoid arthritis exclusion criterion.

^b^Including depression and anxiety.

^c^In total, 2 patients had undergone total colectomy.

^d^Disease extent was classified according to the following hierarchy: pancolitis, left-sided, proctosigmoiditis, proctitis, and other/unspecified.

### Dose Escalation

**Figure 2.**
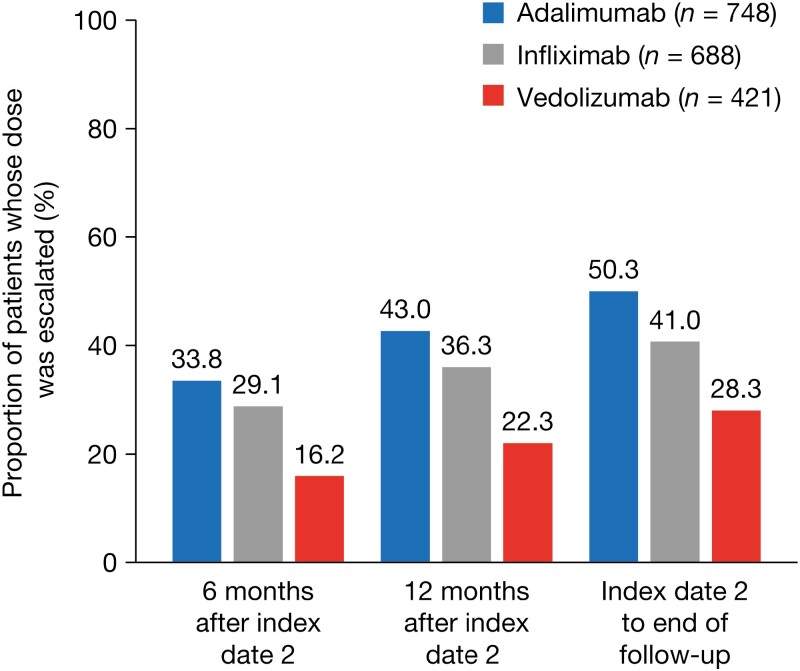
Proportions of patients whose dose was escalated.

**Figure 3.**
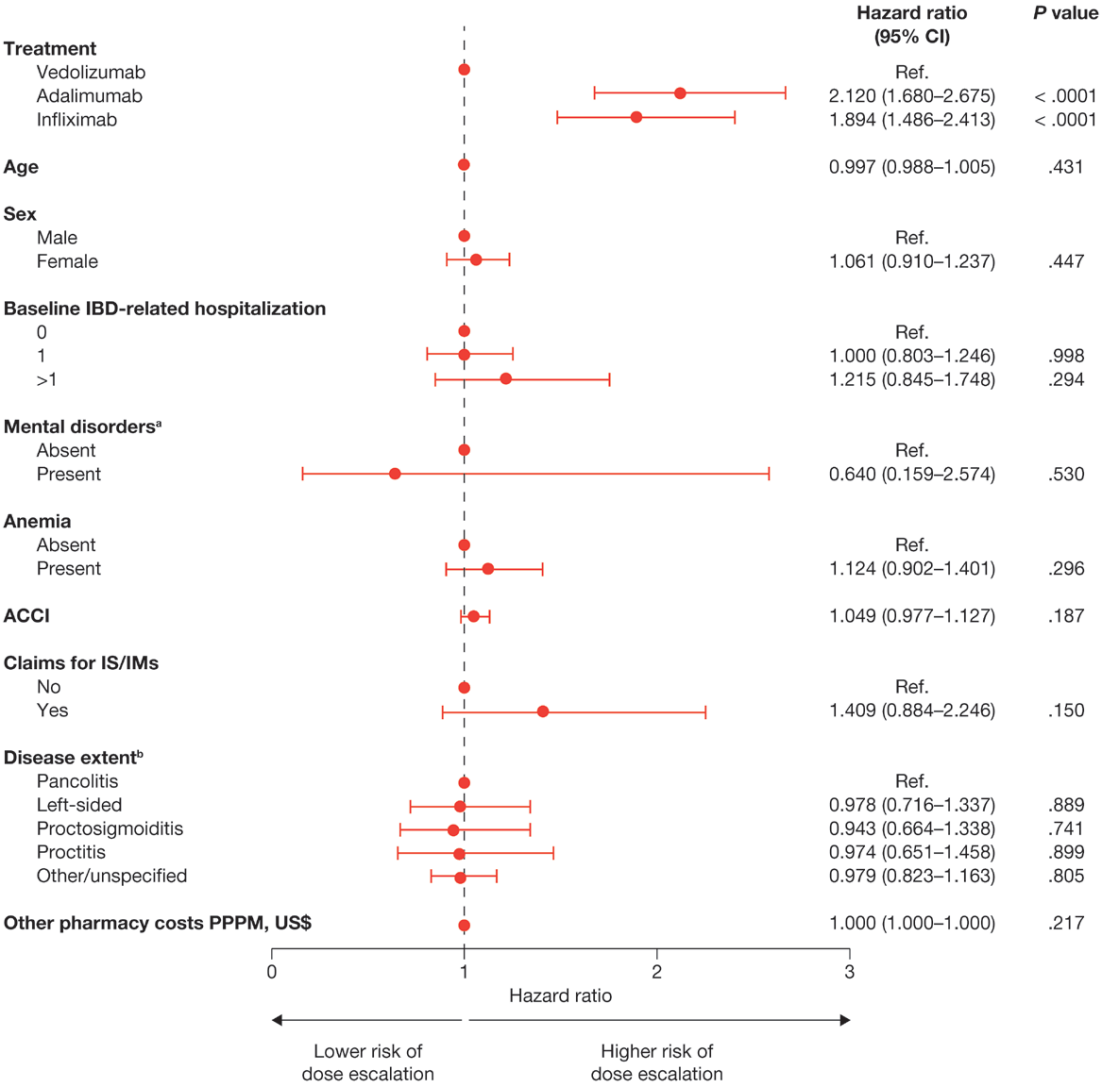
Risk of dose escalation. ^a^Including depression and anxiety. ^b^Disease extent was classified according to the following hierarchy: pancolitis, left-sided, proctosigmoiditis, proctitis, and other/unspecified. ACCI, age-adjusted Charlson Comorbidity Index; CI, confidence interval; IBD, inflammatory bowel disease; IM, immunomodulator; IS, immunosuppressants; PPPM, per patient per month; Ref., reference.

**Figure 4.**
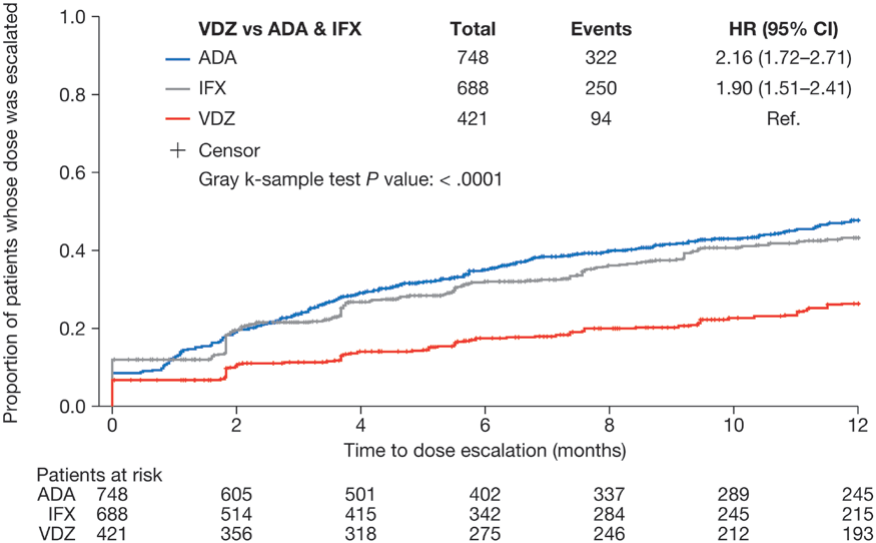
Unadjusted Kaplan–Meier curve for time to dose escalation. ADA, adalimumab; CI, confidence interval; HR, hazard ratio; IFX, infliximab; Ref., reference; VDZ, vedolizumab.

The proportion of patients whose dose was escalated after the initiation of maintenance treatment was lower for vedolizumab than for infliximab or adalimumab at all time points evaluated (Figure 2). The risk of dose escalation was significantly higher for infliximab (hazard ratio [HR], 1.894; *P* <* *.0001) and adalimumab (HR, 2.120; *P* <* *.0001) than for vedolizumab, after adjustment for baseline demographics and clinical characteristics (Figure 3). The time to dose escalation was significantly longer for vedolizumab than for adalimumab and infliximab (*P* < .0001; Figure 4). The HR (95% confidence interval [CI]) for time to dose escalation was 2.16 (1.72–2.71) for adalimumab and 1.90 (1.51–2.41) for infliximab, relative to vedolizumab.

### Infection or Sepsis and IBD-Related Hospitalization

The unadjusted proportions of patients with infection or sepsis or who experienced IBD-related hospitalizations after dose escalation were below 5% for all index drugs (Figure S1). After adjusting for covariates, the odds of experiencing the composite outcome of infection, sepsis, or IBD-related hospitalization after dose escalation were 2 times higher for those who received an anti–TNF-α treatment (adalimumab or infliximab) than for those who received vedolizumab (odds ratio [OR], 2.052; 95% CI, 1.200–3.507; *P* = .009; Figure S2). Patients with 1 baseline IBD-related hospitalization had higher odds of experiencing the composite outcome of infection, sepsis, or IBD-related hospitalization than patients with no baseline IBD-related hospitalizations (OR, 1.817; 95% CI, 1.170–2.822; *P* = .008; Figure S2).

### Corticosteroid Use

The unadjusted proportions of patients receiving corticosteroids after dose escalation were higher for those who received an anti–TNF-α treatment than for those who received vedolizumab (Figure S3). Furthermore, after adjustment for covariates, the odds of corticosteroid use after dose escalation were higher for those who received an anti–TNF-α treatment than for those who received vedolizumab (OR, 1.967; 95% CI, 1.467–2.638; *P* < .0001; Figure S4). The odds of corticosteroid use were predicted to significantly increase with each incremental increase in ACCI score (OR, 1.130; 95% CI, 1.021–1.249; *P *= .018).

### Index Drug Costs

Cost estimates are presented in Table 2. Unweighted mean index drug costs per patient following dose escalation were lower for vedolizumab (US$14,045.44; standard deviation [SD], US$34,871.70) than for infliximab (US$22,284.67; SD, US$50,525.93) and adalimumab (US$39,139.10; SD, US$67,453.71; Table 3). After adjusting for covariates, patients who received an anti–TNF-α treatment were more than 2 times more likely to incur an index drug cost after dose escalation than those who received vedolizumab (OR, 2.295; 95% CI, 1.771–2.975; *P* < .0001; Figure S5). Patients with claims for immunomodulators, immunosuppressive agents, or anti-inflammatory agents during the baseline period had higher odds of incurring an index drug cost than those without claims for these agents (OR, 1.811; 95% CI, 1.041–3.152; *P* = .036). Among patients whose dose was escalated, expected index drug costs after dose escalation were significantly higher for those who received an anti–TNF-α treatment than for those who received vedolizumab (ratio of expected cost, 1.245; 95% CI, 1.029–1.506; *P* = .025; Figure S6). Expected index drug costs were significantly lower for patients with proctosigmoiditis than for those with pancolitis (ratio of expected cost, 0.732; 95% CI, 0.545–0.983; *P* = .038), and for those with anemia than for those without (ratio of expected cost, 0.755; 95% CI, 0.621–0.918; *P* = .005). Expected index drug costs were significantly higher for patients who had a mental disorder than for those without (ratio of expected cost, 3.659; 95% CI, 1.122–11.929; *P* = .032).

**Table 2.** Cost estimates.

Variable	Adalimumab (*n *= 748)	Infliximab (*n *= 688)	Vedolizumab (*n *= 421)
Total index drug induction costs (unweighted),^a^ US$			
Mean, SD	13,319.54 (3475.08)	16,043.60 (13,940.66)	16,061.70 (4599.57)
Median (Q1, Q3)	13,307.78 (12,525.31, 14,194.55)	12,569.26 (9385.18, 17,763.38)	16,444.32 (14,437.34, 18,097.00)
Min, max	(0, 61,485.77)	(0, 134,019.00)	(0, 36,324.64)
Index drug induction costs per patient per month,^a^ US$			
Mean, SD	11,555.14 (4560.16)	4232.04 (3688.56)	4497.38 (1545.20)
Median (Q1, Q3)	12,060.67 (8567.84, 14,555.15)	3401.65 (2487.15, 4736.14)	4732.32 (3799.27, 5419.01)
Min, max	(0, 48,954.98)	(0, 41,368.11)	(0, 11,212.45)
Total other pharmacy costs (unweighted),^b^ US$			
Mean, SD	4524.15 (5391.86)	4335.29 (4826.57)	6376.92 (8561.13)
Median (Q1, Q3)	3457.16 (950.33, 6421.62)	2899.6 (760.47, 6475.97)	4593.85 (1165.11, 8761.25)
Min, max	(0, 87,198.20)	(0, 46,823.48)	(0, 108,755.62)
Other pharmacy costs per patient per month,^b^ US$			
Mean, SD	636.96 (754.00)	445.04 (504.69)	669.72 (902.92)
Median (Q1, Q3)	481.25 (135.45, 909.61)	298.02 (81.14, 668.00)	480.94 (108.59, 936.45)
Min, max	(0, 11,935.50)	(0, 5,113.39)	(0, 11,834.02)
Cost of first maintenance dose, US$			
Mean, SD	2159.03 (356.90)	5503.43 (3822.84)	5849.18 (1334.74)
Median (Q1, Q3)	2209.36 (2105.65, 2340.32)	4626.48 (3578.40, 6044.66)	5954.36 (5502.03, 6354.35)
Min, max	(0, 5,107.30)	(0, 41,531.01)	(12, 14,774.68)
Cost per mg, US$			
Mean, SD	53.98 (8.92)	10.48 (7.28)	19.50 (4.45)
Median (Q1, Q3)	55.23 (52.64, 58.51)	8.81 (6.82, 11.51)	19.85 (18.34, 21.18)
Min, max	(0, 127.68)	(0, 79.11)	(0.04, 49.25)

Abbreviations: max, maximum; min, minimum; Q1, quarter 1; Q3, quarter 3; SD, standard deviation.

^a^Between index date 1 and index date 2 (not inclusive of index date 2).

^b^Six months before index date 1 and index date 2 (not inclusive of index date 2).

**Table 3.** Index drug costs after dose escalation.

Index drug	Overall cohort, *N*	Mean (SD) unweighted drug cost per patient, US$
Adalimumab	748	39,139.10 (67,453.71)
Infliximab	688	22,284.67 (50,525.93)
Vedolizumab	421	14,045.44 (34,871.70)

Index drug	Patients whose dose was escalated, *n*	Mean (SD) cost per patient per month, US$
Adalimumab	322	6207.25 (3297.38)
Infliximab	250	4186.72 (3212.34)
Vedolizumab	94	5026.05 (2436.87)

Abbreviation: SD, standard deviation.

## Discussion

In clinical practice, patients may lose response to their prescribed biologic treatment during maintenance treatment and require dose escalation to recapture response^5^; however, dose escalation can increase healthcare utilization and costs compared with standard dosing,^9^ and may negatively affect other clinical outcomes.^6^ Real-world data are limited, comparing the proportions of patients experiencing adverse outcomes after dose escalation in patients receiving biologics with different mechanisms of action.^6^ This study, therefore, aimed to evaluate the rate of dose escalation among biologic-naïve patients with UC who were treated with vedolizumab or an anti–TNF-α treatment, and to evaluate the impact on adverse outcomes and index drug costs.

In this study, a lower proportion of patients treated with vedolizumab received dose escalation than those treated with adalimumab or infliximab. Furthermore, after adjusting for baseline demographics and clinical characteristics, patients receiving vedolizumab had a significantly lower likelihood of dose escalation than those receiving infliximab or adalimumab. Because dose escalation or adjustment is recommended in the treat-to-target strategy for UC,^16^ these data may indicate greater effectiveness of vedolizumab than anti–TNF-α treatments under the standard dosing regimen. Indeed, in a phase 3 clinical trial evaluating the efficacy of vedolizumab versus adalimumab in patients with moderate-to-severe UC, a higher proportion of patients in the vedolizumab group achieved clinical remission and had endoscopic improvement than the adalimumab group.^17^ However, the rates of dose escalation reported here do not necessarily capture all patients who required dose escalation, which may also be influenced by physician and patient perceptions of increasing dosage as well as drug costs and payer authorization.

Our findings are consistent with the results from a retrospective, longitudinal, claims-based study, which observed that dose escalation, defined as a minimum of 30% increase in the average daily maintenance dose, was more frequent with infliximab and adalimumab than vedolizumab in a mixed population of patients with UC and Crohn’s disease who were either biologic-naïve or experienced.^15^ Similarly, the EVOLVE study’s retrospective chart review of biologic-naïve patients with IBD demonstrated that dose escalation, defined as an increase in the index treatment frequency and/or dose, was less likely for vedolizumab than for anti–TNF-α treatment.^18^ Conversely, a more recent retrospective claims-based cohort study of patients with UC who had not received a biologic 12 months before inclusion in the study, demonstrated that a greater proportion of patients who received vedolizumab or infliximab had a dose escalation than those receiving adalimumab or golimumab, with dose escalation defined as an average daily dose greater than 20% higher than specified on the label.^19^ This study differs from our own, however, in that it did not exclude all patients who received an off-label induction dose. Dose escalation is one outcome that may indicate disease progression or lack of effectiveness of treatment. Other endpoints such as healthcare resource utilization^20^ and disease exacerbations^18^ are also important indicators of real-world treatment effectiveness and should be considered when assessing the clinical and economic implications of UC treatments.

Studies have shown that patients with UC have a greater susceptibility to infections than healthy individuals, due in part to suppression of the immune system that results from treatment with corticosteroids, immunotherapies, and biologics.^21,22^ As such, infection-related hospitalizations, which represent a significant healthcare burden, have been found to be common in patients with IBD.^22^ Additionally, sepsis has been shown to be associated with an increased risk of death in patients with IBD.^22^ We observed that unadjusted proportions of patients who had infection or sepsis were generally low among patients whose dose was escalated, and were lower for vedolizumab than for anti–TNF-α treatment. Furthermore, among patients whose dose was escalated, the adjusted odds of experiencing the composite adverse outcome of infection, sepsis, or IBD-related hospitalization were higher for those treated with an anti–TNF-α treatment (adalimumab or infliximab) than for those treated with vedolizumab. A previous systematic review of dose escalation in UC noted that adverse events after dose escalation were not well reported in the literature, with only 1 study presenting detailed information on the type of adverse event experienced^6,23^; thus, our study presents new and important information on this type of outcome.

We observed that patients who received an anti–TNF-α treatment had increased odds of corticosteroid use after dose escalation compared with those who received vedolizumab. These data suggest that patients who receive vedolizumab are more likely to sustain corticosteroid-free remission, an important guideline-recommended outcome, than those receiving an anti–TNF-α treatment.^2^

In our unadjusted analyses, index drug costs after dose escalation were lowest in patients treated with vedolizumab, while our adjusted analyses demonstrated that patients who received an anti–TNF-α treatment were more likely to incur costs, and that costs after dose escalation were likely to be higher than for those treated with vedolizumab. A recent cost-effectiveness analysis of vedolizumab compared with adalimumab in patients with moderate-to-severe UC, based on the VARSITY trial, demonstrated that patients treated with vedolizumab had lower study drug costs over 2 years than those treated with adalimumab.^24^ Other direct healthcare resource-use-related costs were also lower for vedolizumab than for adalimumab, although this study was not limited to patients whose dose was escalated.^24^

ODESSA-UC presents real-world data on adverse outcomes in biologic-naïve patients with UC after dose escalation. This study included patients who were biologic naïve; thus, these results relate to the use of the index drugs as first-line biologics. We demonstrate that the choice of first-line biologic impacts on the likelihood of receiving dose escalation and subsequent adverse and economic outcomes, as well as the length of time a patient receives treatment before receiving dose escalation. One of the strengths of ODESSA-UC is the use of national databases that include claims data on patients treated at various clinical practices across the United States. In addition, the study provides a robust measurement of dose escalation by capturing both the shortening of dose interval and dose increase of biologic therapies in one metric, with subsequent covariate adjustment for estimating the hazard of dose escalation of each biologic. Furthermore, this study provides insights into outcomes for patients with UCUC after dose escalation that may inform risk–benefit analyses of dose escalation of different biologics. Using the variables identified in this study, future work could include propensity score analysis to further delineate the impact of prior hospitalization, anti-TNFα treatment failure, concomitant immunomodulator use, and comorbid conditions on the risk of dose escalation.

ODESSA-UC is a retrospective observational study and so has some limitations that should be considered when interpreting the results. Despite being a common source of data for observational studies, claims data may be associated with selection and information bias. First, some variables of relevance, such as disease severity, are not available and are likely to impact the risk of dose escalation. Indeed, in a retrospective cohort study of patients with UC receiving vedolizumab, Mayo score at baseline was predictive of subsequent dose escalation.^25^ Although disease severity cannot be directly accounted for in studies of claims data, other clinical characteristics at baseline were captured (such as all-cause and IBD-related hospitalization and use of concomitant medications) and could be considered related to disease severity, and were adjusted for in the models. Similarly, data on the intent to escalate the dose are not recorded in claims databases and this may differ between biologics where authorization approaches are different. Second, medication use was inferred from dispensed prescription records, which may be incomplete or contain errors and cannot be verified. For patient self-administered treatments, compliance and timing of use cannot be verified. Third, in clinical practice, loss of response may be addressed by treatment switching or dose escalation, which may vary between different treatments. However, in this study, patients were censored at the point of treatment switching; therefore, the rate of dose escalation may not be an accurate indicator of loss of response for each index drug. Fourth, there is a risk that patients were lost to follow-up. Owing to the chronic nature of UC and the resultant high-cost burden of treatment, the likelihood of missing claims data is low because it is unlikely that treatments will be paid for without the use of medical insurance. The cohort was also subject to the risk of loss to follow-up as a result of termination of insurance coverage; however, we specified a minimum baseline and follow-up period to reduce the impact of this potential limitation. Fifth, the databases used in our study predominantly include working-age people covered by employer-sponsored private insurance, and thus may not be representative of the general US population. Finally, the inclusion of biologic-naïve patients was based on the absence of a claim for a biologic in the 6-month period before index date 1; thus, patients’ treatment history prior to this period was not considered in these analyses and it is possible that some patients had previously received biologics before the included time frame.

## Conclusions

In conclusion, our real-world study in patients with UC demonstrated differences in the rates of dose escalation, subsequent adverse outcomes, and economic outcomes among first-line biologic therapies, favoring vedolizumab over infliximab and adalimumab. These data may help stakeholders in their understanding of the clinical implications of dose escalation in addition to the associated healthcare resource use.

## Supplementary Material

otad061_suppl_Supplementary_Tables_1-4_Figures_1-6

## Data Availability

Data not publicly available.
