# Supplementary material for: Dose Escalation of Biologics in Biologic-Naïve Patients With Ulcerative Colitis: Outcomes From the ODESSA-UC Study
Source: Crohns Colitis 360. 2023 Nov 16;5(4):otad061. doi: 10.1093/crocol/otad061 (PMC10653026; doi:10.1093/crocol/otad061)
Supplement: otad061_suppl_Supplementary_Tables_1-4_Figures_1-6 [file otad061_suppl_supplementary_tables_1-4_figures_1-6.docx]

# Supplementary Material

## Supplementary Table 1. Diagnostic codes.

| **Diagnosis** | **ICD-9-CM** | **ICD-10-CM** |
| --- | --- | --- |
| Inclusion | | |
| Crohn’s disease | 555, 555.1, 555.2, 555.9 | K50.00, K50.011, K50.012, K50.013, K50.014, K50.018, K50.019, K50.10, K50.111, K50.112, K50.113, K50.114, K50.118, K50.119, K50.80, K50.811, K50.812, K50.813, K50.814, K50.818, K50.819, K50.90, K50.911, K50.912, K50.913, K50.914, K50.918, K50.919 |
| Ulcerative colitis | 556/556.0, 556.1, 556.2, 556.3, 556.5, 556.6, 556.8, 556.9 | K51.00, K51.011, K51.012, K51.013, K51.014, K51.018, K51.019, K51.20, K51.211, K51.212, K51.213, K51.214, K51.218, K51.219, K51.30, K51.311, K51.312, K51.313, K51.314, K51.318, K51.319, K51.50, K51.511, K51.512, K51.513, K51.514, K51.518, K51.519, K51.80, K51.811, K51.812, K51.813, K51.814, K51.818, K51.819, K51.90, K51.911, K51.912, K51.913, K51.914, K51.918, K51.919, K51.31 |
| Exclusion | | |
| Ankylosing spondylitis | 720.0 | M45.x |
| Psoriasis | 696.1 | L40.0–L40.4, L40.8, L40.9 |
| Psoriatic arthritis | 696.0 | L40.5x |
| Rheumatoid arthritis | 714.0 | M05.1–M05.9 or M06 |

Abbreviations: ICD-9-CM, International Classification of Diseases, Clinical Modification, 9th Edition; ICD-10-CM, International Classification of Diseases, Clinical Modification, 10th Edition.

## Supplementary Table 2. Index drug approved dosage and expected daily dose.

| **Study drug** | **Maintenance interval, days** | **Dose** | **Expected daily dose** |
| --- | --- | --- | --- |
| Adalimumab | 28 | 80 mg | 2.9 mg |
| Infliximab | 56 | 5 mg/kg | Varies by weight |
| Vedolizumab | 56 | 300 mg | 5.4 mg |

Based on prescribing information.^1–3^

## Supplementary Table 3. Diagnosis codes for infections of interest and sepsis.

| **Subgroup of infection** | **Diagnosis** | **ICD-10 codes** |
| --- | --- | --- |
| **Opportunistic infections** | | |
| Viral infections | Cytomegalovirus | B25, B27.1 |
|  | Herpes virus | B00–B02, A60.0 |
|  | Epstein-Barr virus | B27.0 |
|  | Progressive multifocal leukoencephalopathy | A81.2 |
|  | Acute viral hepatitis unspeciﬁed | B17.9 |
|  | Viral meningitis | G02.0 |
|  | Viral pneumoniae | J17.1 |
| Mycobacterial infections | Mycobacterial infections | A15–A19, A31 |
| Bacterial infections | Bartonellosis | A44 |
|  | Legionnaires’ disease | A48.1–A48.2 |
|  | Pneumonia and sepsis due to *Streptococcus pneumoniae* | A40.3, J13, B95.3 |
|  | Nocardiosis | A43 |
|  | Actinomycosis | A42 |
|  | Listeriosis | A32 |
|  | Salmonella infections | A02 |
| Fungal infections | Candidiasis | B37 |
|  | Coccidioidomycosis | B38 |
|  | Histoplasmosis | B39 |
|  | Blastomycosis | B40 |
|  | Aspergillosis | B44 |
|  | Cryptococcosis | B45 |
|  | Pneumocystosis | B59 |
|  | Fungal meningitis | G02.1 |
|  | Fungal pneumoniae | J17.2 |
| Parasitic infections | Cryptosporidiosis | A07.2 |
|  | Isosporiasis | A07.3 |
|  | Leishmaniasis | B55 |
|  | Toxoplasmosis | B58 |
|  | Strongyloidiasis | B78 |
| **Serious infections** | | |
| Pulmonary infections | Pneumonia | A48.1, B01.2, B05.2, B25.0, J12–J18, J10–J11 |
|  | Other acute lower respiratory infections | A37, A42.0, B39–B40, B44, B58.3, B59, B95.3, J20–J22 |
|  | Abscessus pulmonis | J85 |
|  | Empyema pleura | J86 |
| Gastrointestinal disease | Intestinal infectious disease | A00–A08, K93.820 |
|  | Viral hepatitis | B15, B17, B25.1 |
|  | Cholangitis | K80, K81, K83.0 K87, B25.8 |
|  | Liver abscess | K75.0 |
|  | Infectious esophagitis | B00.8 |
| Skin and subcutaneous tissue infections | Erysipelas | A46 |
|  | Dermatophytosis and other superﬁcial mycoses | B35–B36 |
|  | Cellulitis and abscess | L02–L03 |
|  | Herpes virus | B00.1–B00.2, B00.7, B00.9, B01.8–B01.9, B02.3–B02.9, B05.3–B05.9, B06.8–B06.9, B08–B09, A60 |
|  | Other local infections of skin, oral tissue, and subcutaneous tissue | A36.3, K11.3–K12.2, L00–L01, L04–L05, L08, L30.3, M72.6 |
| Urinary tract infections | Nephritis | N10 |
|  | Acute prostatitis and prostate abscess | N41.0, N41.2, N41.3 |
|  | Cystitis | N30.0 |
|  | Salpingitis and oophoritis | N70 |
|  | Endometritis | N71.0 |
|  | Cervicitis uteri | N72 |
|  | Syphilis | A50–A53 |
|  | Gonorrhea | A54 |
|  | Chlamydia | A55–A53 |
|  | Orchitis and epididymitis | N45 |
|  | Other urinary tract infections | N39.0, N73.3, N77.1 |
| Ear, nose, and throat infections | Mastoiditis | H70 |
|  | Nasopharyngitis | A36.1 |
|  | Sinusitis | J01 |
|  | Pharyngitis | J02 |
|  | Pharyngeal, retropharyngeal, and parapharyngeal abscess | J36, J39.0–J39.1 |
|  | Tonsillitis | A36.0, J03 |
|  | Laryngitis and tracheitis | A36.2, J04–J05, J37 |
|  | Acute upper respiratory infections of multiple and unspeciﬁed sites | A36.8–A36.9, J06 |
|  | Infection of external ear and acute otitis media | H60.0–H60.3, H65.1–H65.0, H66, H68.0 |
| Musculoskeletal infections | Infectious arthritis | M00–M01 |
|  | Infective myositis | M60.0 |
|  | Osteomyelitis | M86 |
| Other infections | Infection of the eye | B00.5, B30, H00–H01, H10.5, H10.8 |
|  | Infections in the nervous system | A32.1, A39, A80-A89, B00.3–B00.4, B01.0–B01.1, B02.0–B02.2, B05.0–B05.1, B06.0, G00-G02, G04–G07 |
|  | Infections of prosthetic devices, implants, and grafts | T82.6–T82.7, T84.5–T84.7, T85.7 |
|  | Sepsis, systemic inﬂammatory response syndrome of infectious origin, and septic shock | A32.7, A40–A41 |
|  | Certain bacterial disease | A20–A28, A32, A34–A35, A38, A42–A44, A48.0, A48.2–A49.9, B95, B96–B97 |
|  | Spirochetal disease | A65–A69 |
|  | Rickettsiosis | A75–A79 |
|  | Viral infections | A90–A99, B25.2, B25.9, B26–B27; B33–B34 |
|  | Mycoses | B37–B49 |
|  | Protozoal diseases | B50–B57, B58.1–B58.2, B58.8–B58.9, B60–B64 |
|  | Unspeciﬁed infectious diseases | B99.9 |
|  | Acute infective pericarditis and endocarditis | I30.1, I33.0 |
|  | Mycobacterial infections | A15–A19, A31 |

Abbreviation: ICD-10-CM, International Classification of Diseases, Clinical Modification, 10th Edition.

## Supplementary Table 4. Proportions of patients receiving immunomodulators, immunosuppressive agents, anti-inflammatory agents, or other concomitant medication during the baseline period.

| **Medication class** | **Adalimumab**  **(*n* = 748)** | **Infliximab**  **(*n* = 688)** | **Vedolizumab**  **(*n* = 421)** |
| --- | --- | --- | --- |
| 6-mercaptopurine | 61 (8.2) | 58 (8.4) | 36 (8.6) |
| Azathioprine | 748 (100.0) | 109 (15.8) | 71 (16.9) |
| Balsalazide | 600 (80.2) | 451 (65.6) | 278 (66.0) |
| Betamethasone | 527 (70.5) | 477 (69.3) | 255 (60.6) |
| Cyclosporine | 748 (100.0) | 109 (15.8) | 71 (16.9) |
| Leflunomide | 748 (100.0) | 109 (15.8) | 71 (16.9) |
| Lenalidomide | 0 (0.0) | 0 (0.0) | 1 (0.2) |
| Mesalamine | 572 (76.5) | 424 (61.6) | 266 (63.2) |
| Methotrexate | 78 (10.4) | 81 (11.8) | 45 (10.7) |
| Mycophenolate mofetil | 748 (100.0) | 109 (15.8) | 71 (16.9) |
| Olsalazine | 140 (18.7) | 128 (18.6) | 63 (15.0) |
| Sirolimus | 748 (100.0) | 109 (15.8) | 71 (16.9) |
| Steroids | 583 (77.9) | 548 (79.7) | 300 (71.3) |
| Sulfasalazine | 68 (9.1) | 61 (8.9) | 32 (7.6) |
| Tacrolimus | 748 (100.0) | 109 (15.8) | 71 (16.9) |
| Thalidomide | 0 (0.0) | 0 (0.0) | 1 (0.2) |
| Thioguanine | 4 (0.5) | 3 (0.4) | 0 (0.0) |

Data are presented as the number and proportion (*n* [%]) of patients with at least one claim for one of the listed medications at any time during the baseline period. Patients can receive more than one medication. Concomitant medication may be for the treatment of inflammatory bowel disease or for a comorbid condition.

**
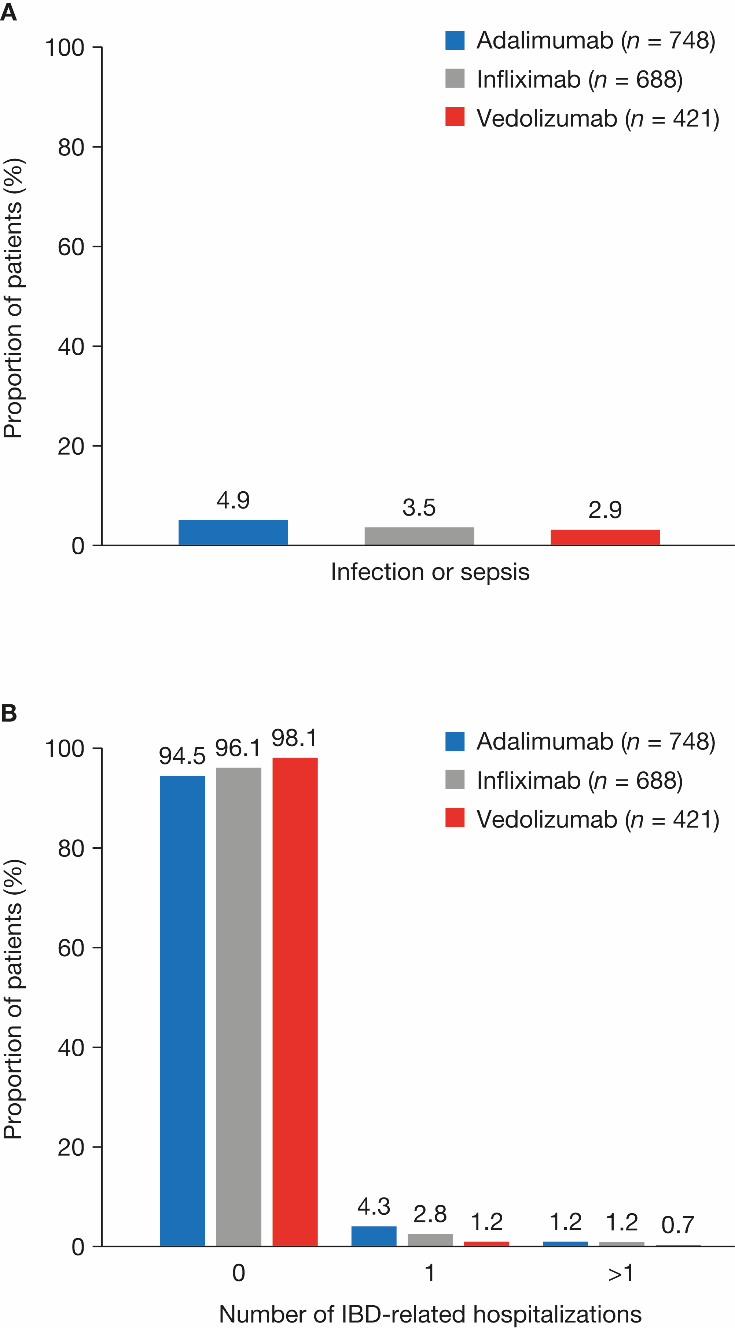
**

## Supplementary Figure 1. Proportions of patients who experienced (A) infection or sepsis, and (B) IBD-related hospitalization after dose escalation. Abbreviation: IBD, inflammatory bowel disease.

##
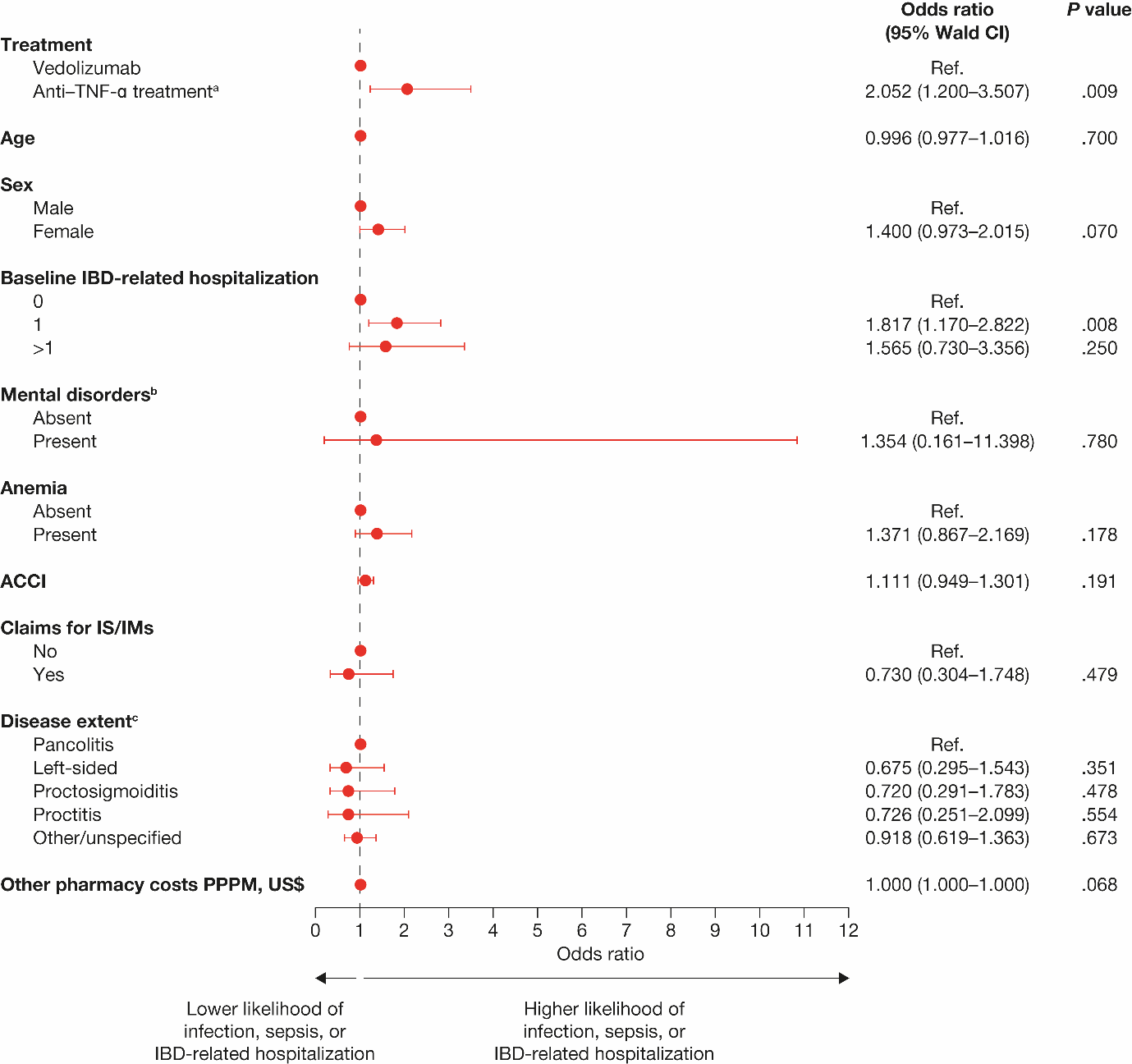
 Supplementary Figure 2. Likelihood of infection, sepsis, or IBD-related hospitalization after dose escalation. ^a^Adalimumab and infliximab. ^b^Including depression and anxiety. ^c^Disease extent was classified according to the following hierarchy: pancolitis, left-sided, proctosigmoiditis, proctitis, and other/unspecified. Abbreviations: ACCI, age-adjusted Charlson Comorbidity Index; CI, confidence interval; IBD, inflammatory bowel disease; IM, immunomodulator; IS, immunosuppressants; PPPM, per patient per month; Ref., reference; TNF-α, tumor necrosis factor-alpha.


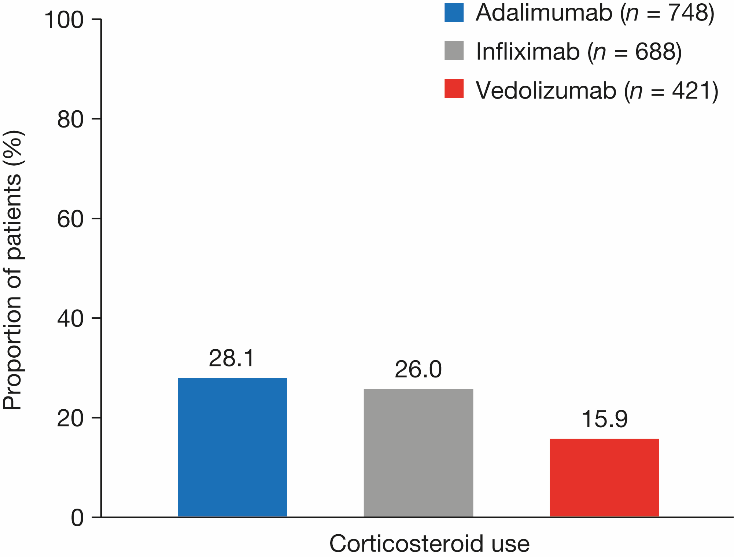


## Supplementary Figure 3. Proportions of patients who used corticosteroids after dose escalation.


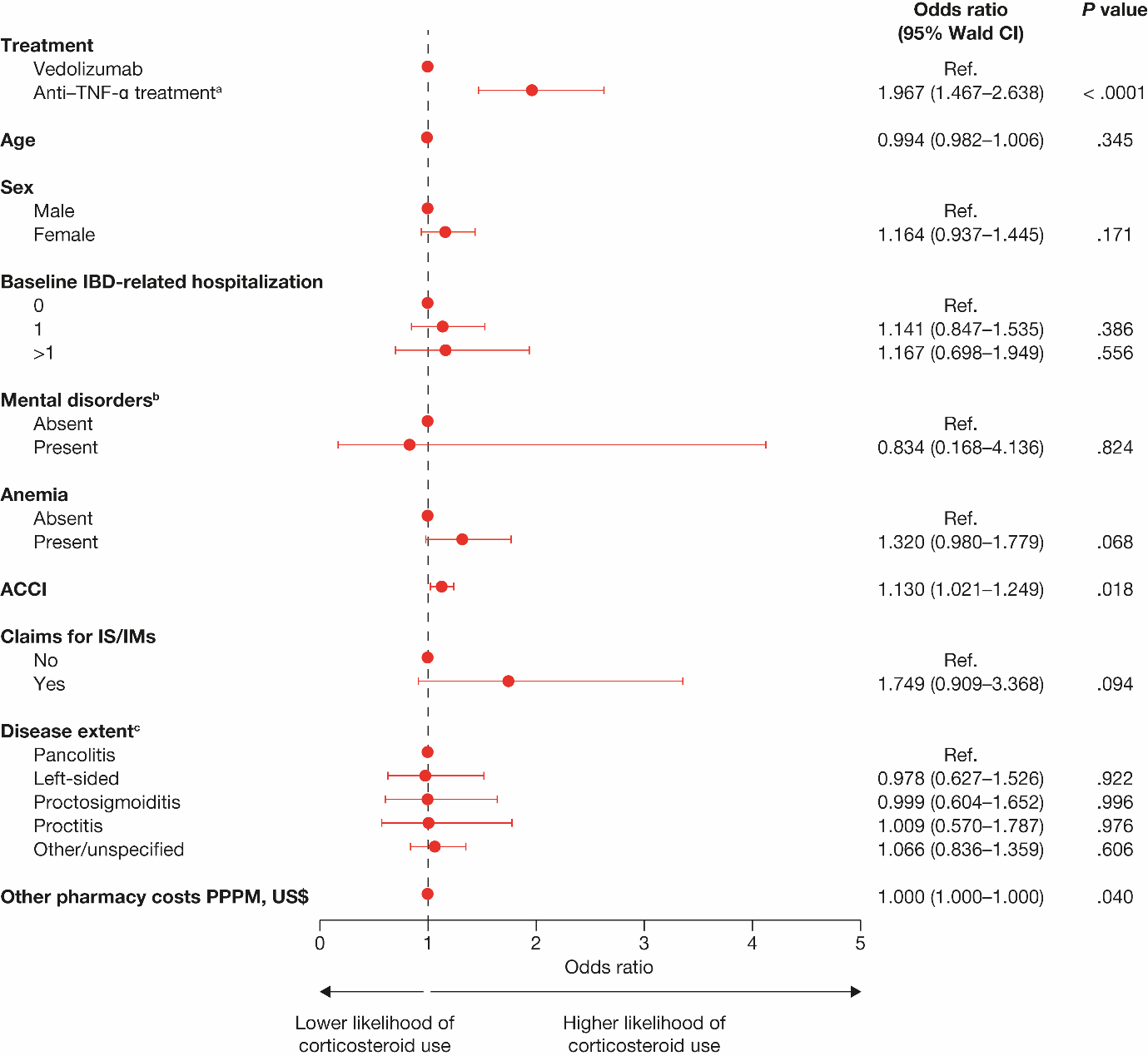


## Supplementary Figure 4. Likelihood of corticosteroid use after dose escalation. ^a^Adalimumab and infliximab. ^b^Including depression and anxiety. ^c^Disease extent was classified according to the following hierarchy: pancolitis, left-sided, proctosigmoiditis, proctitis, and other/unspecified. Abbreviations: ACCI, age-adjusted Charlson Comorbidity Index; CI, confidence interval; IBD, inflammatory bowel disease; IM, immunomodulator; IS, immunosuppressants; PPPM, per patient per month; Ref., reference; TNF-α, tumor necrosis factor-alpha.


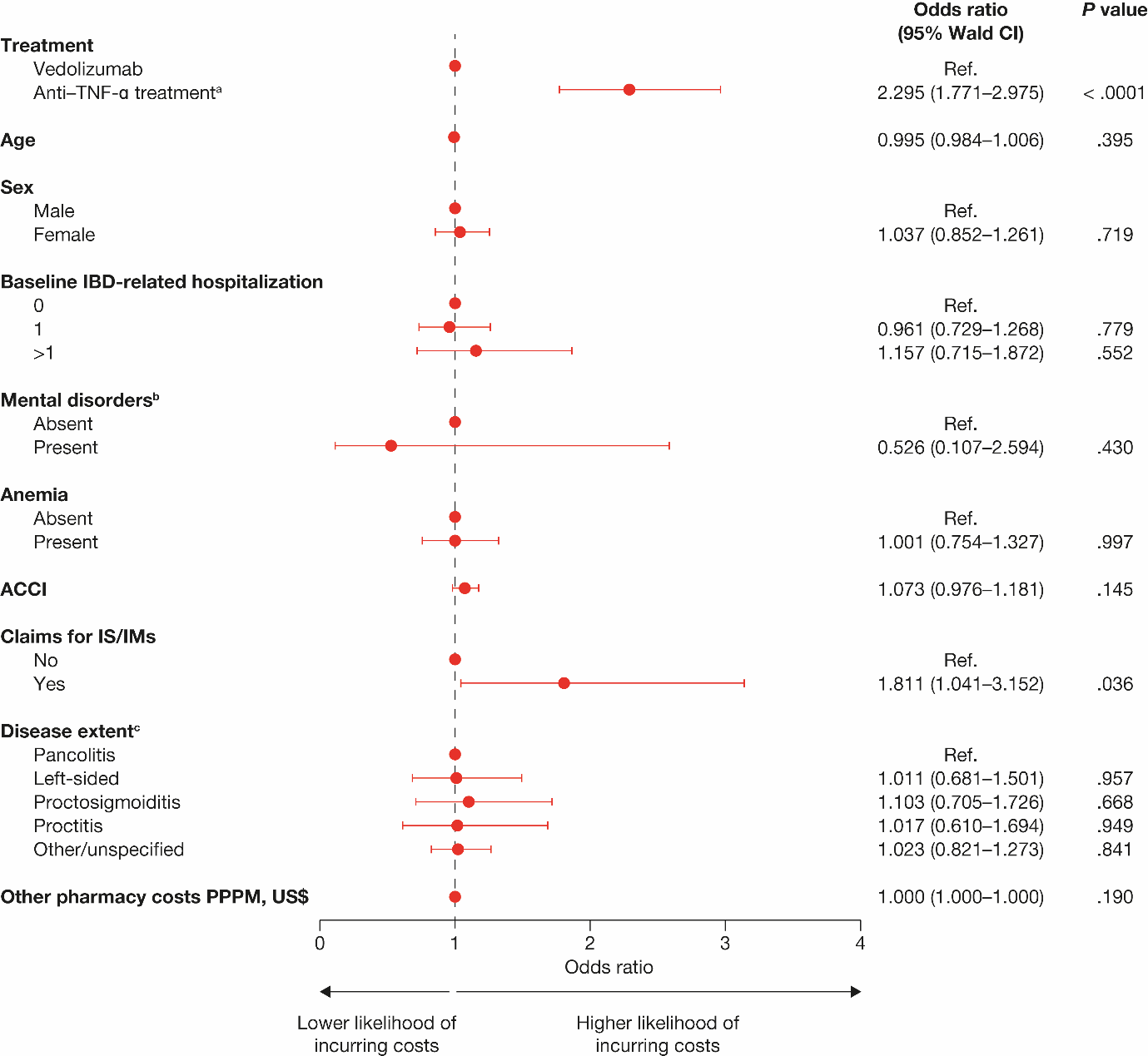


**Supplementary Figure 5.** Likelihood of incurring any index drug costs after dose escalation.
^a^Adalimumab and infliximab. ^b^Including depression and anxiety. ^c^Disease extent was classified according to the following hierarchy: pancolitis, left-sided, proctosigmoiditis, proctitis, and other/unspecified.
Abbreviations: ACCI, age-adjusted Charlson Comorbidity Index; CI, confidence interval; IBD, inflammatory bowel disease; IM, immunomodulator; IS, immunosuppressants; PPPM, per patient per month; Ref., reference; TNF-α, tumor necrosis factor-alpha.


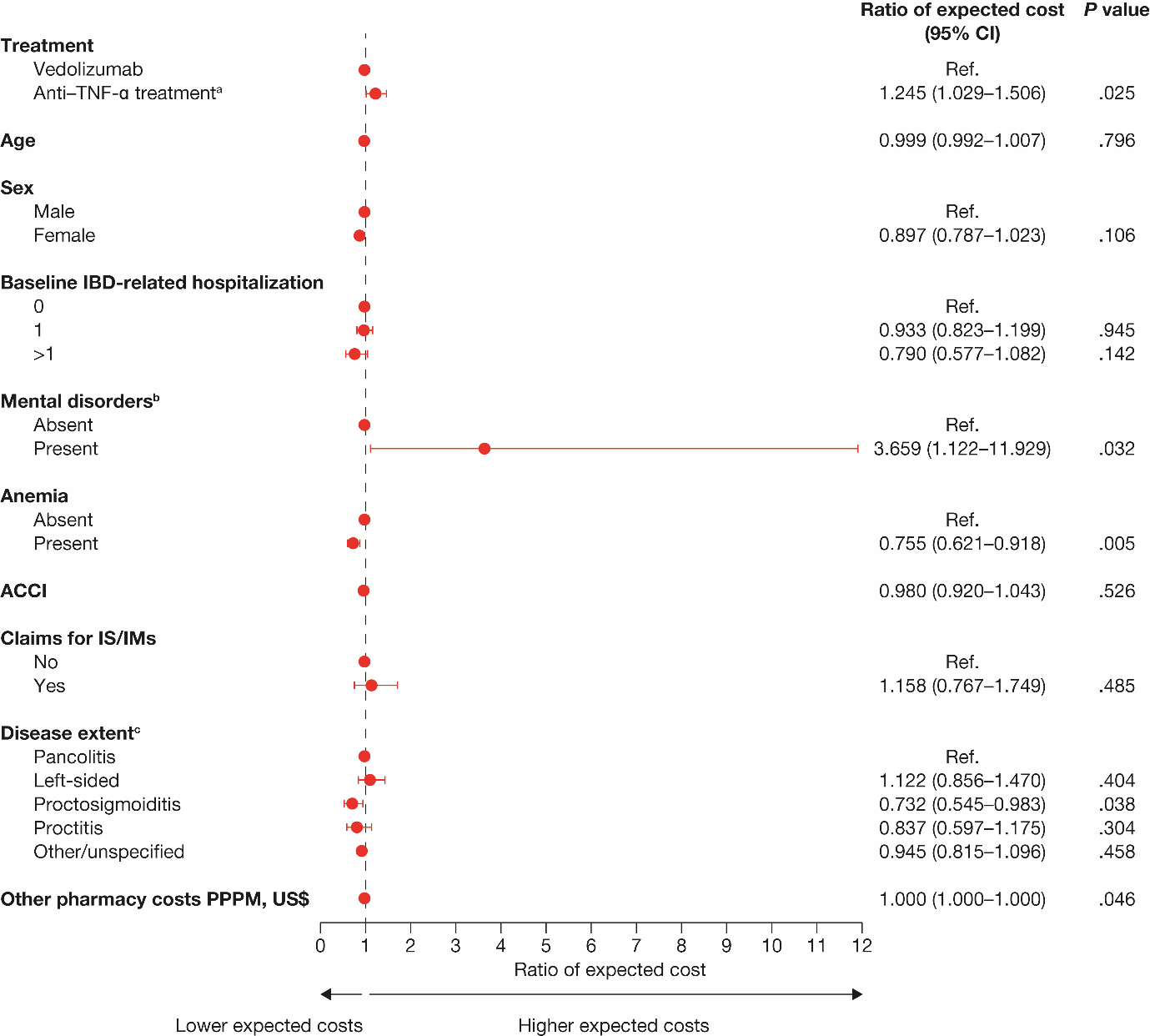


## Supplementary Figure 6. Ratio of expected index drug costs after dose escalation among patients whose dose was escalated. ^a^Adalimumab and infliximab. ^b^Including depression and anxiety. ^c^Disease extent was classified according to the following hierarchy: pancolitis, left-sided, proctosigmoiditis, proctitis, and other/unspecified. Abbreviations: ACCI, age-adjusted Charlson Comorbidity Index; CI, confidence interval; IBD, inflammatory bowel disease; IM, immunomodulator; IS, immunosuppressants; PPPM, per patient per month; Ref., reference; TNF-α, tumor necrosis factor-alpha.

# References

1. AbbVie. https://www.abbviepro.com/gb/en/immunology/dermatology/products/skyrizi/adalimumab-prescribing-information.html. Accessed June 21, 2023.
2. Janssen. https://www.janssenlabels.com/package-insert/product-monograph/prescribing-information/REMICADE-pi.pdf. Accessed June 21, 2023.
3. FDA. https://www.accessdata.fda.gov/drugsatfda_docs/label/2020/125476s025s030lbl.pdf. Accessed June 21, 2023.
